# Supplementary material for: Are carbon emissions trading and green financial instruments synergistic? -Comprehensive quantitative research based on content analysis
Source: PLoS One. 2024 Mar 7;19(3):e0298601. doi: 10.1371/journal.pone.0298601 (PMC10919601; doi:10.1371/journal.pone.0298601)
Supplement: S1 Data — (ZIP) [file pone.0298601.s001.zip › Data/地级市Z_F工作报告.zip!/╦─┤¿/π≥╓▌╩╨/└┤╫╘╣1⁄2╓┌║┼í╛┬φ┐╦╩2╛▌═°í┐_2015.docx]

各位代表：

现在，我代表市人民政府，向大会报告工作，请予审查，并请市政协委员提出意见。

2014年工作回顾

2014年，是我市抢抓机遇、克难奋进的一年。在省委、省政府和市委的坚强领导下，在市人大、市政协的监督支持下，我们紧紧围绕建设川滇黔渝结合部区域中心城市的总体定位，着力稳增长、促改革、调结构、惠民生，经济持续快速发展，社会保持和谐稳定。全市完成地区生产总值1259.7亿元，比2013年（下同）增长11%；规模以上工业增加值增长12.3%；全社会固定资产投资1181亿元，增长36.3%；社会消费品零售总额468.3亿元，增长13.7%；地方一般公共预算收入115.9亿元，同口径增长12.9%；城镇居民人均可支配收入25240元，增长10.6%；农民人均纯收入9470元，增长12%，较好地完成了市七届人大四次会议确定的主要目标任务！向全市人民承诺的民生实事全部兑现！

一、积极应对挑战，经济发展稳中有进

一年来，我们面对宏观经济下行、酒业深度调整、房地产市场变化等多重压力，迎难而上、积极作为，保持了良好的经济运行态势，主要经济指标增速位居全省前列。

综合施策稳定增长。出台了30条稳增长措施，制定了帮助民营企业、中小微企业发展系列政策，积极筹建四川泸州纯粮原酒发展基金，将工业发展引导资金提高到4.8亿元，设立了2亿元医药产业扶持资金，发挥了较好的组合效应。切实帮助区县破解发展难题，促进县域经济加快发展，民营经济实现增加值750.7亿元，增长11.6%。

整合资源优化平台。将全市所有园区整合为四川泸州长江经济开发区、泸州高新技术产业园区、泸州酒业集中发展区“三大园区”。泸州长开区形成“一区五园”布局结构，荣获“四川最具投资价值产业园区”称号；泸州高新区新增建成区面积2052亩，创建国家高新区通过部委会审；泸州酒业集中发展区完成主营业务收入317亿元，被评为“四川省知识产权示范园区”。创新投融资平台，组建了泸州市能源投资有限公司、工业投资集团公司、高新投资公司和交通投资集团公司，投融资渠道有效拓展。

科学规划引领发展。坚持以规划为龙头，编制了《泸州市新型城镇化发展规划（2013—2020年）》、《泸州市综合交通运输体系发展规划》、《泸州港发展战略研究》、《四川泸州长江经济开发区总体规划》、《泸州沱江新城概念性规划》、《泸州长江生态湿地新城概念性规划》、《泸州智慧城市建设概念性规划》、《泸州现代服务业发展规划》、《泸州现代农业发展规划（2014—2025）》、《泸州高新区控制性详细规划》等一系列重大发展规划，完成了第三次全国经济普查登记，启动了“十三五”规划编制工作。

二、加快转型升级，八大产业稳步发展

一年来，我们围绕“产业发展突破年”的工作主题，大力改造提升传统优势产业，加快培育发展新兴产业，八大产业实现增加值1025亿元。

工业结构调整步伐加快。坚持优先发展酒业不动摇，加快调整产品结构，“工农牌”、“歪嘴郎”、“泸小二”等产品销售市场不断拓展。大力发展纯粮固态酿造，种植高粱70.1万亩。泸州老窖“一园三基地”、中国酒镇·酒庄、郎酒浓香型白酒生产基地、华夏龙窖白酒产业园等项目建设顺利推进。全市规模以上酒类制造业增加值增长12.1%。化工、能源、机械产业转型升级取得积极成效。年产15万吨润滑油加氢项目开工建设，煤制气一期工程、北方公司纤维素醚生产线等项目加快推进。观文煤矿投入试运行；页岩气累计产气1亿立方米；北辰电力产业园建设有序推进；成功列入全国第二批新能源汽车推广应用示范城市。四川长液·厦门海翼西部基础件项目建成投产。现代医药产业快速发展，阿斯特年产50万套医疗器械、科瑞德药物制剂生产线等项目建成投产，成功引进山东步长集团等医药企业9家、总投资70亿元。全市规模以上医药制造业增加值增长17.7%。

现代服务业快速发展。加快发展商贸物流业，城西城市综合体、西南商贸中心二期等项目建设有序推进，成功举办“第二届中国（泸州）西南商品博览会”；“拉货宝”、“泸州购”等本土电子商务平台全线运营，全市电子商务实现交易额90亿元、增长39.8%；泸州港保税物流中心（B型）成功获批，泸州水运口岸成为国家第一批进境粮食指定口岸，首批从澳大利亚进口的粮食顺利抵达泸州港，新开通“泸州至南京至韩国”近洋航线；泸州港完成货物吞吐量3134万吨、增长15.8%，其中：集装箱吞吐量32万标箱、增长59%；泸州机场通航航线达14条，实现旅客吞吐量71万人次、增长61.6%。加快发展现代金融业，乐山银行在我市设立分行；四川宏图物流公司在“新三板”成功挂牌上市。2014年末，银行业金融机构各项存款余额1613亿元、贷款余额920.8亿元，较年初分别增长14.3%和20.1%。加快发展旅游业，张坝桂圆林、天仙硐创建为国家4A级景区，成功创建全省旅游标准化示范市，实现旅游总收入183.9亿元、增长28.6%。大力发展健康养老、文化创意等新兴服务业，我市成功列入全国首批养老服务业综合改革试点城市。

现代农业稳步推进。大力发展特色农业产业，实施121个产业化重点项目，完成投资88.2亿元。成功举办“泸州优质农产品（北京）展销周活动”。粮食总产量达198.3万吨，实现稳中有增。肉类总产量达34.4万吨、增长3.3%。竹林面积达351万亩，居全省第一。新增省级标准化示范项目8个、省级龙头企业5家，新发展农民合作社示范社33个、家庭农场220个。主要农作物耕种收综合机械化水平达40%。锁口水库、观文水库、倒流河水库等骨干水利工程建设加快推进，建成高标准基本农田41万亩，新增耕地2.9万亩、节水灌面15.7万亩。幸福美丽新村建设扎实推进，新建聚居点70个。我市成功创建为国家现代农业示范区。

三、狠抓项目建设，发展后劲不断增强

一年来，我们强力实施项目攻坚，全社会固定资产投资总量居全省第3位，比2013年提升4位。

重点项目加快推进。实施207个重点项目，完成投资820.2亿元。成自泸赤高速公路泸州段建成通车，全市高速公路通车里程达378公里，居川南第一位。93公里绕城高速公路闭合运行。泸州机场迁建工程、叙大铁路、叙古高速公路、叙宜高速公路泸州段、泸合产城大道、纳福港城大道等项目建设加快推进。隆黄铁路叙永至毕节段预可研获国家发改委批复。武骏特种玻璃制品生产线建成投产，泸州新能源新材料产业园、四川合辰实业循环经济等项目开工建设。

要素保障持续有力。全年投资到位资金1204亿元，增长31.2%。民间投资881.9亿元，增长32.4%。争取各类用地指标2.9万亩。关口110千伏、泸县220千伏等一批输变电工程建成投运。工业用电量39.5亿千瓦时、增长4.2%，工业用气量12.6亿立方米、增长10%，成品油供应量48.1万吨、增长13.1%。

抢抓机遇成效明显。主动对接国家建设长江经济带发展战略，被列为全国重要区域性综合交通枢纽（节点城市），一批重大项目列入长江经济带发展规划。利用全国资源枯竭城市转型发展、乌蒙山片区区域发展与扶贫攻坚等政策机遇，积极向上争取项目、资金支持。储备重大项目480个、总投资9000亿元。

四、完善城镇体系，区域中心城市建设步伐加快

一年来，我们围绕建设区域中心城市，加快推进新型城镇化进程，全市城镇化率达44.8%。房地产开发完成投资192.6亿元、增长47.1%；建筑业实现总产值518.9亿元、增长27.3%。

中心城区功能品质进一步提升。启动“两江新城”规划建设，实施76个重点项目，完成投资74.3亿元。沱江六桥、忠山隧道、云龙机场快速通道等项目建设加快推进，改造完成立体交通3处，建成人行天桥5座。城东、城南等污水处理厂及截污干管工程有序推进，新建、改造供水管网25公里。“两江四岸”整治工程完成长江现代城段和沱江滨江路景观改造。中心城区建成区面积达113.2平方公里、人口达116.6万人。成功创建国家园林城市。

县城和小城镇建设加快推进。泸县、合江、叙永、古蔺四个县城实施城市建设重点项目38个，完成投资66.1亿元，建成区面积增加3.4平方公里，人口增加3.5万人。全市新增国家级重点镇13个、省级“百镇建设试点行动”示范镇10个、中国历史文化名镇（村）3个。

争取国家新型城镇化综合试点取得成功。积极探索试点工作，出台了《泸州市新型城镇化综合试点工作方案》。全面放开落户限制，引导和鼓励农业转移人口20278人落户城镇。5个乡撤乡设镇，改设或增设街道办事处5个。我市成为全省唯一的国家新型城镇化综合试点地级市。

五、推进改革创新，发展动力有效激发

一年来，我们坚持改革与创新并举，着力增强发展内生动力，在重点领域实施了62项具体改革事项。

政府职能加快转变。积极推进政府机构改革，市政府工作部门由33个调整为32个。调整、下放行政审批事项131项，取消行政事业性收费23项。全面推行“两集中三到位一杜绝”，办结行政审批和公共服务事项175万件。在全国率先启动了不动产统一登记示范建设工作。在全省率先开展工商登记“先照后证”试点，新增各类市场主体2.8万户、各类企业5200户，分别增长21.7%和85.7%。

经济体制改革加快推进。稳步推进泸天化集团改革工作，华西证券有限责任公司改制为股份有限公司，兴泸水务集团启动股份制改造工作。市商业银行增资扩股进展顺利，四川中国白酒产品交易中心申请第三方支付牌照工作有序推进，启动了纳溪、泸县农村信用社改制工作。加强财政预算管理制度建设，调整市区财政管理体制，实现了市区税收属地征管。农村产权制度改革加快推进，农村集体土地所有权、集体建设用地使用权、宅基地使用权确权登记工作基本完成。

创新能力加快提升。大力推进科技创新平台建设，泸州高新区创新创业服务中心创建成省级科技企业孵化器，国家酒类包装产品质量监督检验中心通过资质认定和授权，四川省高性能液压件工程技术研究中心启动建设。设立科研专项资金4000万元，与科研院校开展合作项目249个。新增国家高新技术企业8家，转化重大科技成果52项，实现高新技术产值100亿元、增长18%。我市成功创建为全国质量强市示范城市。

六、扩大开放合作，区域竞争力不断增强

一年来，我们坚持“走出去”与“引进来”相结合，开放空间有效拓展，发展优势明显增强。

开放水平进一步提升。成功承办“2014中国国际酒业博览会”，12个国家和地区的420多家企业参展，成交金额128.2亿元。“中国国际酒业博览会”落户泸州。我市成为第十五届西博会主题市。成功举办“四川（泸州）-法国医药健康产业国际合作交流会”。与新加坡开放合作三年规划加快落实，与杭州国际城研中心等单位的战略合作不断深化。全市实际到位外资增长19.1%，进出口总额增长21.7%。

招商引资取得实效。全市新履约市外国内项目679个，投资总额1128.3亿元。积极参加“中外知名企业四川行”、“万企出国门”、科博会等活动，成功举办“两江新城”推介会、中国酒城·泸州投资说明会。新引进投资10亿元以上的项目20个。

区域合作不断深化。加强长江沿岸城市交流合作，与南京市政府、上海国际港务集团签署港口物流发展战略合作协议。积极推动赤水河区域合作发展，与遵义、毕节、昭通等市签署战略合作框架协议。主动融入成渝经济区，推动川南经济区一体化发展。实施对口援建甘孜州稻城县、乡城县，雅安市宝兴县项目17个，完成投资1.1亿元。

七、持续改善民生，社会事业全面发展

一年来，我们始终把保障和改善民生作为工作的出发点和落脚点，加强基层基础建设，促进了社会和谐稳定。

民生工程有力推进。全市民生支出196亿元，占一般公共预算支出的70%。城镇新增就业39956人，城镇登记失业率3.14%。建立了统一的城乡居民基本养老、基本医疗和大病保险制度。开展法律援助23447人次。开工建设保障性住房6985套，启动实施棚户区改造26191套、农村危房改造14405户。整治“三无院落”113个。新改建农村公路973.1公里。地质灾害避险搬迁1868户。深入推进“七大扶贫攻坚行动”，帮助7.6万人脱贫解困。解决了农村30.5万人饮水安全问题和12.9万户用电难问题，全面消除了“无电校”和“无电户”。

社会事业协调发展。深入实施素质教育，促进教育事业均衡发展，新建、改扩建中小学校16所，新建公办幼儿园19所，“上学难、入园贵、大班额”等问题得到进一步化解，教育质量稳步提升，出台政策解决了农村偏远教师生活补贴问题。川滇黔渝结合部医教园区建设加快推进。泸州医学院更名四川医科大学通过教育部高校设置评议委员会评议。卫生事业加快发展，西南医疗康健中心、市人民医院新院等项目建设顺利推进，市妇幼保健院强化了临床支撑。9家县级公立医院综合改革全面推进。在叙永县3个乡镇开展了全民免费预防保健试点工作。启动实施“单独两孩”政策，审批6636例。文体广电事业蓬勃发展，话剧《蒋兆和》在北京、成都等地成功演出，《天赐泸州》首次公演，4个区（县）、5个乡镇成为“全省民间文化艺术之乡”，泸县创建为全国文化先进县。奥林匹克体育公园建成投用。参加省第十二届运动会夺得44枚金牌，居全省第三名，创历史新高。

生态建设进一步加强。强化节能减排，化学需氧量、氨氮、二氧化硫、氮氧化物分别削减1.5%、1.8%、24%、28%，大气可吸入颗粒物（PM10）下降5.1%。水生态文明城市建设试点工作扎实推进，在全省率先出台最严格的水资源管理制度。泸州垃圾焚烧发电厂建设加快推进。投入资金6574万元，实施赤水河流域泸州段环境综合整治。全市新建乡镇污水处理厂26个。治理水土流失面积107.5平方公里，完成营造林48万亩，巩固退耕还林61.5万亩。

社会保持安全稳定。认真落实安全生产“党政同责、一岗双责、齐抓共管”责任制，严格实行“一票否决”。安全生产“四项指标”全面下降，我市成为全省未发生较大以上安全生产事故的两个市之一。落实了食品安全“五级监管”制度，食品药品安全监管工作不断强化。集中开展打黑除恶专项斗争暨社会治安专项整治行动，破获各类刑事案件7534起。大力推进社会治安防控体系建设，中心城区“天网”三期工程建成投用。坚持用群众工作统揽信访工作，依法规范信访秩序，调解各类矛盾纠纷16485件，社会大局保持了和谐稳定。

国防动员、人民防空、民兵预备役建设不断加强。拓展双拥共建领域，海军592舰被命名为“泸州舰”。妇女儿童、青少年、慈善、残疾人、老龄事业持续发展，民族宗教、台湾事务、外事、侨务、消防、保密、档案、气象、地方志等工作取得了新成绩。

各位代表，过去一年，我们以开展党的群众路线教育实践活动为契机，切实加强政府自身建设，深入查找“四风”突出问题，扎实开展“9+1”专项整治，“三公”经费支出减少14.8%。认真落实依法治市工作规划，依法行政进一步加强。严格执行市人大及其常委会的各项决议决定，自觉接受市人大、市政协的监督，办理人大代表建议和政协委员提案376件、办结率100%。坚持政府常务会会前学法，调整完善市政府法律顾问团，严格执行规范性文件合法性审查和“三重一大”民主决策制度。深化政务公开，建成行政权力依法规范公开运行系统，网上公开运行行政权力事项近3万件。全面推进政府投资项目工程招投标和政府采购电子化。“12345”市长热线受理群众有效来电2.9万件。认真落实党风廉政建设责任制，切实加强行政监察、财政监督和审计监督，廉政风险防控“三早三卡”机制进一步完善。严厉查处了政府系统195起违纪案件，始终保持了惩治腐败的高压态势！

各位代表，过去一年，成绩来之不易。这是省委、省政府和市委正确领导的结果，是市人大、市政协和社会各界监督支持的结果，是全市人民共同努力的结果。在此，我代表市人民政府，向给予我们监督支持的人大代表、政协委员、各民主党派、工商联、无党派人士和各人民团体，向驻泸部队官兵和社会各界人士，向全市人民，表示衷心感谢并致以崇高敬意！

各位代表，总结过去一年的工作，我们也清醒看到，发展中还存在不少困难和问题：一是加快经济发展和结构调整升级的双重任务仍然艰巨。白酒、化工、能源、机械等传统优势产业还处于深度调整期；现代医药、高端装备制造、新能源新材料等新兴产业虽然发展较快，但总量偏小，短期内对经济的支撑作用不明显。二是重点领域和关键环节改革还需加快推进，妨碍发展的体制机制障碍亟待破除。三是关系群众切身利益的教育、医疗、扶贫、安全生产、社会治安等方面，还存在不少问题。四是政府自身建设还存在薄弱环节，一些政府工作人员“庸懒散浮拖软”和“不具体、不落实、不负责”等不良现象还时有发生，依法行政、廉洁从政等工作还需进一步加强。对这些困难和问题，我们将采取有力措施积极解决。

2015年工作安排

今年是“十二五”规划的收官之年，是全面深化改革、加快转型发展的关键一年。做好今年政府工作，要认真学习贯彻习近平总书记系列重要讲话精神，主动适应新常态，主动作为稳增长，以创先争优的志气、攻坚克难的勇气、开拓创新的锐气，抢抓国家建设长江经济带等机遇，坚持“稳中求进、进中求好、好中求快”的工作基调，突出“产业转型发展年”的工作主题，向改革要动力、向开放要活力、向创新要效益，把规划变为行动，把蓝图变成现实，加快建设全国重要区域性综合交通枢纽，加快建设长江上游产业转型升级示范区，加快建设川滇黔渝结合部区域中心城市，奋力开创泸州经济社会持续健康发展新局面。

全市经济社会发展的主要预期目标是：地区生产总值比2014年（下同）增长9%，规模以上工业增加值增长10.5%，全社会固定资产投资增长15%，社会消费品零售总额增长10.5%，地方一般公共预算收入增长9%，城镇居民人均可支配收入增长9.5%，农民人均纯收入增长11%。

围绕上述目标，重点做好以下工作：

一、推动产业转型发展，加快构建现代产业体系

加快调整优化白酒产业。坚持夯实基础、调整结构、拓展市场，支持泸州老窖、郎酒等优势酒类企业创新营销模式，扩大市场份额；加大对“小巨人”企业的扶持力度，引导中小微酒类企业整合壮大。大力发展纯粮固态酿造，高粱种植面积稳定在70万亩，打造“泸州酿”区域品牌。有序推进泸州老窖“一园三基地”、郎酒浓香型白酒生产基地等项目建设。充分发挥国家酒检中心等功能平台的作用，确保国家酒类包装产品质量监督检验中心通过验收并投入运行。办好“2015中国国际酒业博览会”。

加快发展现代农业。以国家现代农业示范区建设为抓手，积极推进现代农业示范片、示范点建设。大力发展现代养殖、高效林竹、绿色蔬菜、精品果业等八大特色农业产业，加强农产品品牌建设。严守耕地红线，耕地保有量达到38.4万公顷以上。确保粮食总产量达195万吨以上，优质稻面积稳定在180万亩，优质蔬菜、名优水果面积分别达到100万亩以上；肉类总产量达到36.2万吨以上。新建现代林业基地20万亩。提高主要农作物耕种收综合机械化水平2个百分点。新增市级龙头企业15家，创建国家农民合作社示范社8个，发展家庭农场40个。开工建设泸县三星桥水库等抗旱水源工程，加快推进叙永纳坪、古蔺朝门烟区水源工程建设，不断完善农业基础设施。

加快发展现代制造业。大力推进化工、能源、机械产业转型升级。确保煤制气一期、年产15万吨润滑油加氢等项目建成投产。支持中海沥青提升炼油产能，推动石油化工产业做大做强。开工建设中海石油气电集团生物质天然气项目，确保北辰电力产业园项目建成投产，推进西部智能电网装备产业园、航天泸州工业园等项目建设。加快建设岔角滩煤矿、箭竹坪煤矿。完成长起、邦立重机、长江机械一期项目搬迁，确保泸州长江机械有限公司“智能工厂”建成投产。大力发展现代医药产业，开工建设步长生物制药及新药产业化基地、泸州食品药品检测中心、泸州医学成果转化中心、秦巴GAP研究中心等项目，办好“2015年中国医药企业家年会”。

加快发展现代服务业。推进华润购物中心、海吉星农产品商贸物流园一期、万诚国际红星美凯龙商城、步步高购物中心等项目建设。加快建设泸州临港物流园，建成进出口商品展示交易中心，启动跨境电商工作。办好“第三届中国（泸州）西南商品博览会”、迎春购物月等活动。推动“邻家壹佰”智慧社区超市建设，推进电子商务进农村试点工作。积极筹建泸州农村商业银行，完成泸县农村信用社改制工作。积极引进股份制银行在我市设立分支机构，支持泸州市商业银行在成都设立办事处，推进企业境内外上市，加快证券、保险、债券业发展，促进小额贷款公司、融资性担保公司规范健康发展。强化区域合作，开展旅游营销，着力打造“醉游中国”白酒金三角旅游线、“长征丰碑”红色旅游线，力争龙桥文化生态园、尧坝古镇、花田酒地创建为国家4A级景区。大力培育健康养老、文化创意等新兴服务业。促进房地产业持续健康发展。

加快建设“三大园区”。泸州长开区突出港口功能完善和区域物流合作，大力发展先进制造业和现代物流业，积极争创省级开发区。泸州高新区突出项目带动、开放支撑和体制保障，加快建设长江经济带创新发展示范区，争创为国家高新区。泸州酒业集中发展区突出功能完善和品质提升，完成主营业务收入300亿元以上。

二、提升互联互通能力，加快建设区域性综合交通枢纽

推进交通基础设施建设。加快建设对外运输通道，力争叙古高速公路叙永至古蔺县城段建成通车，推进叙宜高速公路泸州段、泸渝（G8515）高速公路建设，做好叙威高速公路项目前期工作；加快建设叙大铁路，开工建设川南城际铁路泸州段，力争隆黄铁路叙永至毕节段开工，积极开展渝昆铁路、沿江铁路前期工作；加快建设泸州机场迁建工程；协调推进长江泸渝段航道险滩整治。加快建设市内干线路网，推进泸合产城大道、纳福港城大道建设，改造提升重要产业基地、重点城镇、重点景区林区道路；新改建通乡公路150公里、通村公路200公里。完成城北公交枢纽站主体工程，加快城西客运中心站、叙永川泸西外客运站等项目建设。

推进交通运输服务体系建设。依托高速公路，优化客运布局，提升客运服务水平。稳步推进城乡客运一体化建设，逐步开行城乡公交车，促进农村客运区域化经营。优化城市公交体系，促进城市缓堵保畅。力争泸州机场开通新航线，旅客吞吐量突破80万人次。加快建设泸州港续建工程，完善港口集疏运体系，集装箱吞吐量突破38万标箱。鼓励运输企业向现代物流企业转型，积极争创国家4A、5A级物流企业。

推进交通运输支持系统建设。加快建设“智慧交通”，完成泸州港综合信息平台一期工程建设，开发“智慧交通”手机APP功能，初步形成交通大数据与云平台中心。加快建设“绿色交通”，大力推广应用新能源汽车，加大淘汰“黄标车”和老旧车力度。加快建设“平安交通”，建成交通运输日常监测与应急指挥中心，完善交通运输运行监控体系和车辆、船舶GPS及3G视频监控功能，确保交通安全。

三、提高新型城镇化水平，加快建设区域中心城市

加快中心城区建设。坚持“有保有压、突出重点、有序推进、健康发展”，推进城市建设。完成“两江新城”分区规划，优化调整控制性详细规划。沱江新城完成西南商贸中心二期、蜀泸大道四期工程建设，加快建设沱江六桥及连接线、川滇黔渝结合部医教园区、西南医疗康健中心一期、城西城市综合体等项目。长江生态湿地新城建成欢乐派海滩公园，加快建设麒麟组团等项目，启动长江湿地生态公园一期工程建设。坚持“疏解老城、完善功能、提升品质”，改造提升老城区。完成忠山隧道工程建设。建成城东、城南污水处理厂及截污干管工程。推进枇杷沟历史文化街区征收安置、蓝田旧城改造、麻沙桥-学士山综合开发等项目建设。深入开展爱国卫生运动，确保通过第六次“国家卫生城市”复审。加快建设“智慧城市”。

推进县城和小城镇建设。指导四个县城提升规划、建设和管理水平，推动泸县、合江与中心城区一体化发展，辐射带动叙永、古蔺县城建设，增强县城综合承载能力和吸纳能力。着力建设一批基础条件好、发展潜力大、示范作用强的重点集镇，力争2—3个镇列入全国建制镇示范试点镇。重点围绕“两线两点一基地一龙头”，加快建设幸福美丽新村，全面推进农村危房改造及困难户、无房户农房建设。

开展新型城镇化综合试点工作。积极探索农业转移人口市民化成本分担机制，设立专项基金，建立社会保险衔接制度，完善公共服务体系。探索多元化可持续的城镇化投融资机制，促进筹资渠道多元化。创新行政管理高效与行政成本降低的管理模式，启动泸县撤县设区工作，支持合江加快撤县设市进程，有序推进乡镇区划调整。

四、深化改革开放，增强发展动力活力

推进重点领域和关键环节改革。加快转变政府职能，进一步简政放权，加大政府向社会购买公共服务力度。实施事业单位分类改革、公务用车制度改革。深入推进国有企业改革，完善现代企业制度，推进国有经营性资产集中统一监管，推动泸天化集团深化改革、转型发展。深化投融资体制改革，规范政府投资项目，加强融资平台建设和管理，鼓励社会资本通过PPP等方式参与项目建设。继续实施工商登记“先照后证”试点。加快财税体制改革，进一步规范预算管理和政府性债务管理。深化农业农村体制改革，加快农村产权确权颁证，推进土地承包经营权流转。争取并做好土地制度综合改革试点工作，探索推进农村宅基地制度改革。继续推进不动产统一登记示范建设工作。激活民间资本活力，加强企业家能力建设，助推民营经济转型升级，促进民营经济健康发展。

加强创新能力建设。大力推进科技创新，实施50个科技成果转化项目，新增国家高新技术企业8户、省级创新型企业8户。培育和发展科技服务业，推进科技孵化平台建设，确保泸州高新区综合孵化器建成投运，加快医药等专业孵化器建设。优化提升公共技术服务平台，加快“川南科技成果与专利技术实体交易市场”建设，启动高端装备研究院、知识产权服务中心建设。积极争创国家知识产权试点城市。实施“酒城科技英才”计划，培养、引进一批重点产业高层次、高技能人才。实施质量升级计划，深入推进质量强市工作。

深化对外开放合作。注重招大引强，围绕重点产业、重点园区，立足全球市场，开展产业链、供应链、价值链招商，千方百计引进大企业、大集团、大项目。泸州长开区重点招引一批有实力的商贸物流、现代制造企业，力争中国物流城市配送中心、公路货运中心、生产资料流通中心等项目签约落地。泸州高新区重点围绕高端装备制造、新能源新材料、现代医药产业链，力争引进一批重点项目。深化与沿江港口城市的合作，密切与成渝经济区城市的协作，推动川南经济区一体化进程。加强与滇东、黔北地区的对接，加快赤水河区域合作发展。继续深化与新加坡的交流合作。

五、着力保障和改善民生，促进发展成果全民共享

加大民生投入力度。确保公共财政用于民生支出的比重保持在70%以上。全面落实各项就业促进政策，确保城镇新增就业3.5万人，城镇登记失业率控制在4.5%以内。完善基本养老和社会救助制度体系，推进机关事业单位工作人员养老保险制度改革。进一步扩大社保覆盖面，提高保障水平。推进医疗卫生服务体系建设，在叙永县开展全民免费预防保健试点工作。加强城市应急备用水源工程建设。解决农村29.7万人饮水安全问题。开展农村集中式饮用水源整治。改造城镇危旧房和棚户区16072套，新开工保障性住房1940套。推进“宽带乡村”惠民工程建设，解决农村“上网难”问题。加大地质灾害防治力度，提升地质灾害综合防治水平。强化风险监测和日常监管，提升食品药品安全监管能力。

加大扶贫攻坚力度。继续以乌蒙山片区为主战场，大力开展精准扶贫，深入推进“七大扶贫攻坚行动”，重点扶持56个贫困村脱贫解困，减少贫困人口7万人以上。加快贫困地区、边远山区基础设施建设，完善叙永、古蔺农电体制改革，推进农村电网改造。开展“扶贫结对网络行”等活动，动员更多社会力量参与扶贫。

加大生态建设力度。巩固退耕还林成果，实施新一轮退耕还林、天然林资源保护工程，加快国家木材战略林基地建设。推进石漠化综合治理、矿山地质环境恢复、土壤污染修复等工程建设，治理水土流失面积120平方公里。加快水生态文明城市建设，确保通过国家中期评估。大力整治农村环境污染，推进城乡污水、垃圾处理设施建设。确保泸州垃圾焚烧发电厂一期建成投用。深入开展大气、水污染综合防治，扎实推进节能减排工作，全面完成总量减排目标任务。

六、加强社会建设，保持社会和谐稳定

大力发展社会事业。深化教育综合改革，推动教育均衡发展，加快中高职衔接和职业院校集团化建设步伐，推进国家中小学教育质量综合评价改革试验区工作，全面提升教育教学质量。加快构建现代公共文化服务体系，开展“醉美泸州·百姓舞台”等文体惠民活动。推进宋代石刻博物馆、文化艺术中心、白招牌全民健身广场、广播电视“户户通”、固定电影放映点等项目建设。加快基本公共卫生服务项目建设，提升公共卫生服务均等化水平。加强重大疾病防控。加快市人民医院新院、市中医院新院建设，完成市妇幼保健院二期工程前期工作。人口自然增长率控制在6.6‰以内。加强国防动员、国防后备力量和民兵预备役建设。做好双拥、民族宗教、老龄、妇女儿童、残疾人、对台、外事、侨务、保密、档案、气象、人防、地方志等工作。

强化安全生产监管。认真贯彻落实新修订的《安全生产法》，强化政府监管责任和企业主体责任。加快推进安全生产标准化建设。强化煤矿和非煤矿山、消防、危化品、公共场所等重点行业、重点领域的安全生产监管。着力夯实安全生产基层基础，建成1个国家级、8个省级安全社区，新建一批省级、市级安全文化示范企业。动态排查治理安全隐患，持续开展“打非治违”专项斗争，坚决防止发生重特大安全生产事故，千方百计确保人民群众生命财产安全。

加强和创新社会治理。加强城乡基层组织建设，充分发挥村（居）民自治作用。健全维护群众权益和化解纠纷机制，加强专业化和行业性人民调解组织建设，及时化解各类矛盾纠纷。进一步畅通和拓宽群众诉求表达渠道，依法规范信访秩序。深化社会治安专项集中整治，严厉打击违法犯罪行为，健全立体化防控体系，促进社会治安大局持续平稳向好。强化信息研判和动态预警防范，提高维稳处突水平。启动戒毒康复医院建设，强化社区矫正工作，切实加强特殊人群管理。强化应急管理，提高防灾减灾能力。全面落实依法治市工作规划，完善社会普法教育机制，扎实开展“法律七进”活动。

七、深入推进依法行政，加快建设法治政府

依法全面履行政府职能。推进政府机构、职能、权限、程序、责任法定化，建立并向社会公布政府权力清单、责任清单和市场准入负面清单。深化行政审批制度改革，全面清理、取消非行政许可审批事项。规范前置审批和中介服务事项，制定投资项目审批核准前置要件目录，取消无法定依据的中介服务事项。加强市、县、乡三级政务服务标准化建设，强化政务服务和公共资源交易平台一体化管理。

健全依法决策机制。完善《重大行政决策程序规定》，把公众参与、专家论证、风险评估、合法性审查、集体讨论决定确定为重大行政决策法定程序。在全市行政机关全面推行法律顾问制度。建立行政决策终身责任追究制度及责任倒查机制。

推进行政执法体制改革。积极推行综合执法，大幅减少市县两级政府执法队伍种类，加强行政执法队伍建设。推行行政复议委员会制度。健全完善城管执法体制，推进执法事权重心下移，全面推进城市管理相对集中行政处罚权工作。严格规范行政执法程序和行政执法裁量权，向社会公开发布裁量标准。加大关系群众切身利益的重点领域执法力度，依法惩处各类违法行为。

强化行政权力制约监督。严格执行市人大的决议决定，自觉接受市人大、市政协的监督，切实做好建议提案办理工作。自觉接受司法监督、舆论监督和社会监督，拓宽群众监督渠道，及时回应处理群众合法合理诉求。全面推进政务公开，切实加强财政预算、重大项目建设、公益事业建设等领域的政府信息公开，建立全市行政规范性文件查询检索系统。强化政府内部权力制约，加强对财政资金分配使用、公共资源转让、政府投资、政府采购等重点领域监管。

加强作风和廉政建设。巩固党的群众路线教育实践活动成果，建立健全密切联系群众、改进作风长效机制，持续治理“四风”、“三不”、“庸懒散浮拖软”等不良现象。严格落实党风廉政建设责任制，完善廉政风险防控“三早三卡”机制。加强廉政宣传教育，强化理想信念、宗旨观念、廉洁自律和警示教育，促进政府工作人员廉洁从政。建立行政监察与审计监督联动机制，严格监督执纪问责，严肃查办腐败案件。

各位代表，新的一年，新的征程。主动适应经济发展新常态，做好今年政府各项工作，任务艰巨、责任重大。让我们在市委的坚强领导下，在市人大、市政协的监督支持下，紧紧依靠全市人民，凝心聚力、锐意进取、扎实工作，为泸州实现“两个率先”而努力奋斗！
